# Advances in Neoantigen-Based Cancer Vaccines

**DOI:** 10.3390/cancers18010144

**Published:** 2025-12-31

**Authors:** An-Chih Wu, Yusuke Nakamura, Kazuma Kiyotani

**Affiliations:** 1 Laboratory of Immunogenomics, Center for Intractable Diseases and ImmunoGenomics (CiDIG), National Institutes of Biomedical Innovation, Health and Nutrition (NIBN), Ibaraki-shi, Osaka 567-0085, Japan; 2 Graduate Institute of Medical Sciences, College of Medicine, Taipei Medical University, Taipei 11031, Taiwan

**Keywords:** cancer vaccine, personalized neoantigen, shared neoantigen, T cell therapy, vaccine platforms

## Abstract

Neoantigen-based immunotherapies harness somatic mutations as tumor-specific targets and represent a major advance in personalized cancer treatment. Since neoantigens are presented exclusively on cancer cells, they enable highly selective T-cell recognition with minimal off-tumor toxicity. Neoantigen vaccines are rapidly emerging as a versatile class of personalized cancer immunotherapies designed to prime tumor-specific T cells by targeting somatic mutations unique to each patient’s tumor. Multiple types of neoantigen vaccines, using peptide, mRNA, and DNA, have shown feasibility, safety, and immunogenicity across diverse solid tumors. Emerging comparative data indicate that the vaccines using peptide-pulsed dendritic cells (DCs) elicit higher per-epitope CD8^+^ T cell responses than mRNA-based vaccines, likely due to more efficient class I presentation of synthetic peptides and ex vivo-loaded DCs. In contrast, mRNAs, despite their capacity of targeting multiple neoantigen peptides simultaneously, often induce CD4^+^-dominant responses due to immunodominance patterns during antigen processing. Recent clinical trials in melanoma, glioblastoma, pancreatic cancer, and other types of cancer have demonstrated not only robust immune activation but also encouraging relapse-free outcomes when administered in adjuvant settings. Treatment timing strongly influenced immune responsiveness; patients with early-stage disease or those vaccinated after surgical resection generally exhibit more preserved systemic immunity and greater vaccine-induced T cell expansion compared to those with advanced disease. Future progress will rely on improved neoantigen prediction, including incorporation of post-translationally modified antigenic targets and acceleration of manufacturing pipelines to ensure timely, personalized vaccine delivery. Collectively, neoantigen vaccines offer substantial promise for integration into next-generation cancer treatment strategies.

## Simple Summary

Cancer-specific neoantigens have become highly attractive targets for cancer immunotherapy. Neoantigen-targeted vaccines aim to activate specific T-cell responses by targeting mutations unique to an individual patient’s tumor. Recent clinical trials of peptide-, mRNA-, and DNA-based neoantigen vaccines have demonstrated both feasibility and safety across multiple cancer types. Among these platforms, peptide-pulsed dendritic cells consistently elicit higher per-epitope CD8^+^ T-cell response rates, likely because they directly deliver defined peptides that are efficiently presented on HLA class I molecules. In contrast, RNA-based vaccines offer advantages in targeting multiple neoantigen epitopes but often generate more CD4^+^-dominant responses. Importantly, neoantigen vaccines induce stronger immune responses in earlier treatment settings, when immune function in patients is relatively preserved. As technological advances accelerate antigen prediction and manufacturing, neoantigen vaccines are becoming a key component for personalized cancer immunotherapy.

## 1. Introduction

Immune checkpoint inhibitors (ICIs) have transformed the therapeutic landscape of multiple cancers and are now established alongside surgery, chemotherapy, and radiotherapy as a standard therapeutic modality. Despite these achievements, only 10–30% of patients experience durable benefit from ICIs, primarily due to insufficient tumor-specific immune activation. This limitation is especially evident in immunologically “cold” tumors, which are characterized by sparse T cell infiltration and strong immunosuppression. These challenges underscore the need for therapeutic approaches capable of reliably inducing potent and highly tumor-specific T cell responses.

Cancer neoantigens, peptide epitopes derived from cancer-specific somatic mutations, have emerged as highly promising targets for next-generation cancer immunotherapies. Since neoantigens are absent in normal tissues, they are recognized as non-self by the host immune system, enabling selective tumor targeting with minimal autoimmune toxicity. Advances in next-generation sequencing, computational algorithms, and immunopeptidomics have accelerated the identification of both personalized neoantigens unique to individual patients and shared neoantigens arising from recurrent driver mutations.

These scientific advances have driven the rapid clinical development of multiple neoantigen-targeted cancer vaccines, which enhance endogenous antitumor immunity by priming neoantigen-specific T cells in vivo. A wide range of vaccine platforms, using peptides, mRNA or DNA, have been tested in early-phase clinical trials. Notably, the global development of mRNA vaccine for SARS-CoV-2 has further accelerated technological innovation and manufacturing capacity in this field, facilitating its broader application to personalized cancer vaccines. These neoantigen-targeted vaccines are considered to induce strong cancer-specific immune responses compared to earlier cancer vaccine strategies, such as whole-cell tumor vaccines or DC-tumor fusion vaccines targeting a broad repertoire of tumor-associated antigens. However, each vaccine type has distinct advantages and limitations with respect to immunogenicity, scalability, and logistical feasibility.

In parallel, adoptive T cell therapies such as tumor-infiltrating lymphocyte and T cell receptor (TCR)-engineered T (TCR-T) cell therapies, have also been explored as complementary neoantigen-directed approaches. While these modalities offer the potential to deliver high numbers of neoantigen-specific T cells, their clinical application remains limited due to challenges in cell manufacturing, scalability, and patient selection.

In this review, we focus primarily on neoantigen vaccine strategies and summarize current clinical evidence across solid tumors as well as comparative evaluations of different vaccine platforms.

## 2. Neoantigens

Neoantigens are cancer-specific antigens derived from somatic alterations that occur in cancer cells and are presented on human leukocyte antigen (HLA) molecules. These neoantigen-HLA complexes are recognized by TCRs, serving as specific markers for T cells to identify cancer cells [1]. Since neoantigens are absent in normal tissues, they are highly attractive molecular targets for immunotherapies. In addition, the immunological feature of neoantigens is related to thymic central tolerance. During thymic T-cell development, negative selection eliminates cells that strongly recognize self-antigens expressed in the thymic microenvironment. Because neoantigens are generally absent from the antigen repertoire involved in central tolerance induction. As a result, T cells recognizing neoantigens presented on HLA largely escape thymic negative selection and are therefore maintained in peripheral T-cell repertoire with relatively high functional avidity [1]. Importantly, escape from central tolerance does not imply the absence of downstream regulatory constraints, as neoantigen-specific T cells remain subject to peripheral tolerance mechanisms, functional exhaustion, and tumor-induced immunosuppression, particularly in advanced disease settings.

Neoantigens can be broadly categorized into two groups, personalized and shared. Personalized neoantigens originate from patient-specific mutations in tumors and therefore require individualized computational prediction based on HLA types of each patient as well as individualized manufacturing processes. While therapies targeting personalized neoantigens can, in principle, be applied to any patient harboring the relevant mutations and maintaining expression of HLA molecules, their selection and production require substantial time and resources. In contrast, shared neoantigens, which are derived from recurrent driver gene mutations, are present across many tumors although their presentation is still limited to patients’ HLA molecules. Those epitopes, such as derived from KRAS, TP53, PIK3CA, FGFR3, or APC, can be applied as standardized, off-the-shelf therapeutic products to a subset of defined patient populations [2,3,4].

Despite their clear conceptual and translational appeal, the identification of neoantigens does not necessarily translate into immunogenicity in vivo. The probability that a predicted neoantigen elicits measurable T-cell responses in vivo is shaped by multiple variables, including mutation clonality, transcriptional and translational expression, efficiency of antigen processing, peptide presentation on HLA, and the existence of responsive T-cell precursors. Consequently, immune response rates reported in clinical studies should be interpreted as probabilistic outcomes influenced by both tumor-intrinsic and host-related factors rather than deterministic consequences of epitope selection. The therapeutic relevance of neoantigens is therefore defined not only by their tumor specificity but also by their translational potential forming the conceptual framework for the neoantigen-directed immunotherapies discussed in subsequent sections.

## 3. Neoantigen-Targeted Cancer Vaccines

Neoantigen-based cancer vaccines aim to prime or boost tumor-specific T-cell responses by presenting predicted neoepitopes to the immune system. Because many neoantigens elicit de novo CD4^+^ and CD8^+^ responses, these vaccines can reshape anti-tumor immunity in cancer patients and are likely to complement existing immunotherapies. We discuss clinical studies evaluating both personalized and shared neoantigens below.

### 3.1. Personalized Vaccines

Personalized vaccines have produced robust evidence of feasibility, safety, and immunogenicity. Recent clinical studies evaluating personalized neoantigens are summarized in Table 1.

One of the earliest clinical studies of neoantigen-targeted therapy was reported for melanoma in 2015. In this report, autologous dendritic cells (DCs) loaded with neoantigen peptides were administered and 33.3% of neoantigen peptides expanded both pre-existing and de novo neoantigen-reactive CD8^+^ T cells [5]. In a melanoma study using multi-peptide vaccine reported in 2017, 6 patients vaccinated with up to 20 neoantigen peptides developed strong CD8^+^ responses (15.5% of the total peptides used in the study) and CD4^+^ responses (59.8%), with durable disease control and successful clinical response by PD-1 blockade upon relapse [6]. Similar results have been reported in multiple solid tumors [9,10].

In glioblastoma, despite its highly immunosuppressive microenvironment, two early trials showed clear biological activity. The vaccine induced polyfunctional CD8^+^ and CD4^+^ T cells (at 5.4% and 13.5% of the neoantigens, respectively) and suggested migration of neoantigen-specific T cells into glioblastoma in steroid-free patients [7]. Hilf et al. reported a median progression-free survival (PFS) of 14.5 months and a median overall survival (OS) of 24.8 months, exceeding outcomes expected with standard therapy [8]. Importantly, neoantigen-specific T-cell responses were detected in both CD8^+^ and CD4^+^ compartments; peptide-specific response rates were 38.5% for CD8^+^ T cells and 84.6% for CD4^+^ T cells. These observations were confirmed in a large cohort of 173 patients with glioblastoma, where vaccine-specific T-cell responses were observed in 87 (90%) of 97 patients and median OS from the first diagnosis reached 31.9 months [15].

Evidence of durable clinical control has also emerged in other malignancies. In a clinical study for clear-cell renal cell carcinoma, no recurrences were reported at 40.2 months post-vaccination, and the vaccine elicited durable tumor-reactive T-cell responses with minimal toxicity [17]. In advanced non-small lung cancer (NSCLC) cases receiving first-line pembrolizumab and chemotherapy, the addition of the personalized long-peptide vaccine yielded a 69% objective response rate (ORR) and a median PFS of 7.2 months, accompanied by vaccine-induced CD8^+^ T-cell responses in 31% of the peptides and CD4^+^ T-cell responses in 39% of the peptides [13].

Several studies including ours reported that DCs loaded with personalized neoantigen peptides can generate measurable immune responses and achieve extended survival, particularly when disease biology allows sufficient time for immune priming [12,16]. More recent clinical evidence supports similar feasibility and immunogenicity in difficult-to-treat cancers [18]. In resectable pancreatic cancer, neoantigen peptide-pulsed DC infusion led to detectable neoantigen-specific immunity in 81.3% of treated patients (29.4% of CD8^+^ and 25.0% of CD4^+^ T-cell responses), accompanied by a favorable clinical course in a substantial proportion of individuals. Collectively, vaccines using neoantigen peptides reliably induce broad T-cell responses and epitope spreading, particularly in the adjuvant setting.

Vaccines using mRNA encoding tandemly concatenated multiple neoantigen epitopes have shown potent immunogenicity with favorable safety across multiple tumor types. In 2017, vaccines using personalized mRNA induced polyfunctional CD8^+^ and CD4^+^ T-cell responses in 24.8% and 48.0% of the vaccinated neoantigens and led to tumor regression or prolonged stable disease condition [19]. More definitive clinical benefit was confirmed in the phase IIb KEYNOTE-942 trial, in which 157 patients with high-risk resected melanoma received personalized neoantigen mRNA vaccine with pembrolizumab (*N* = 107) or pembrolizumab alone (*N* = 50). The neoantigen mRNA vaccine significantly improved relapse-free survival (18-month RFS: 78.6% vs. 62.2%; HR 0.56, *p* = 0.026), with a manageable safety profile, primarily grade 1–2 adverse events [21].

In pancreatic cancer, a tumor type with low baseline immunogenicity, an individualized mRNA neoantigen vaccine induced neoantigen-specific CD8^+^ T cells in 8 (50%) of 16 resected patients [20]. Notably, all eight responders remained recurrence-free at a median follow-up of 18 months, compared with 2 of 8 immune non-responders (median RFS of 13.4 months, *p* = 0.003). In this study, grade 3 adverse events were observed in only one patient, consisting of fever and hypertension. In extended follow-up, 6 of the 8 responders remained recurrence-free at approximately 3 years, consistent with the persistence of long-lived neoantigen-specific CD8^+^ T-cell clones observed up to 3 years post-vaccination [25].

Although less clinically mature, DNA-based neoantigen vaccines have shown meaningful early signs of immunogenicity and disease control across solid and hematologic malignancies. In triple-negative breast cancer, these vaccines induced CD8^+^ T-cell responses in 22.7% of treated peptides and 78% of 18 treated patients and a promising long-term RFS (3-year RFS rate of 87.5%) [23]. Notably, many of the clinical observations described above were derived from early-phase and/or single-arm studies that were not powered for definitive efficacy endpoints; therefore, further examined and validated in larger-scale and randomized clinical trials will be required to clearly delineate their clinical significance and eliminate potential confounding by baseline disease and immune conditions.

### 3.2. Shared Neoantigen Vaccines

Shared neoantigen vaccines target recurrent driver mutations, enabling broader applicability while retaining mutation specificity. Although still in early clinical development, several studies have provided proof-of-concept that even highly conserved oncogenic mutations can serve as effective immune targets (Table 2).

In diffuse midline glioma, H3.3K27M-targeted peptide vaccine induced neoantigen-specific CD8^+^ T-cell responses in 39% of patients, associated with improved OS (median 16.1 vs. 9.8 months for non-responders), with no grade ≥3 toxicities [26]. Similarly, IDH1 R132H peptide vaccination elicited polyfunctional CD4^+^ T-cell responses in 93.3% of glioma patients accompanied by pronounced immune infiltration and favorable long-term disease control [27]. In mismatch-repair-deficient (MSI-H/dMMR) cancers, vaccines using recurrent frameshift peptides induced immune responses in 86.3% of patients and significantly reduced metachronous tumors [28]. More broadly, a pan-cancer platform targeting KRAS and TP53 mutations induced neoantigen-specific T cells in 88% and 83% of evaluable cases for KRAS and TP53, respectively [29]. Collectively, these studies underscore that shared neoantigen vaccines can function as potent, safe, and broadly applicable immunotherapeutic modalities across diverse malignancies.

Beyond vaccine-based approaches, shared neoantigens are also attractive targets for adoptive T-cell therapies. Early clinical studies have demonstrated that T-cell receptors recognizing shared neoantigens can mediate potent and mutation-specific antitumor activity [30,31]. As complementary strategies to vaccines, shared neoantigens targeted TCR-T-cell therapies have been investigated under early-phase clinical trials [32,33]. A landmark study reported complete and durable regression in metastatic pancreatic cancer following infusion of KRAS G12D/HLA-C08:02-specific TCR-T cells. Another study demonstrated a partial response in metastatic epithelial cancer using TP53 R175H/HLA-A02:01-specific TCR-T cells. In addition to TCR-T cell therapies, bispecific antibodies targeting shared KRAS or TP53 neoantigens are also reported although these were still under preclinical studies [34,35]. These shared neoantigen-targeted strategies may facilitate the development of off-the-shelf therapeutic products for selected patient populations.

**Table 2:** Published clinical studies of shared neoantigen vaccines.

Author	Cancer Type	Vaccine Type	Number of Patients	Treatment Setting	Target Antigens	Combination Therapy	Immune Responses	Clinical Outcomes
Mueller, 2020 [26]	Glioma	Long peptide	29	Adjuvant	H3.3K27M	-	Neoantigen-specific T-cell responses in 39% of patients	12-mo OS 40%; mOS 16.1 vs. 9.8 mo in immune responders vs. non-responders; four grade 3 AEs
Platten, 2021 [27]	Glioma	Long peptide	32	Adjuvant	IDH1 R132H	Standard therapy	Neoantigen-specific CD4^+^ T-cell responses in 93.3% patients	3-yr PFS 63%; 3-yr OS 84%; no vaccine-related grade 3/4 AEs
Kloor, 2020 [28]	MSI-H/dMMR cancers	Long peptide	22	Adjuvant	TAF1B, HT001, AIM2	-	Neoantigen-specific T-cell responses in 86.3% patients	One SD > 7-mo no vaccine-related grade 3/4 AEs
Rappaport, 2024 [29]	Pan-cancer	Self-amplifying mRNA	19	Advanced	KRAS G12C, G12D, G12V, G13D, Q61H TP53 R213L, S127Y	Nivolumab + Ipilimumab	KRAS neoantigen-specific T-cell responses in 88% patients, TP53 neoantigen-specific T-cell responses in 83% patients	ORR 0%; SD 42%; PD 58%; mPFS 1.9 mo and OS 7.9 mo; two grade 3/4 AEs
Wainber, 2025 [36]	Pancreatic cancer, colorectal cancer	Long peptide	25	Adjuvant	KRAS G12D, G12R	-	Neoantigen-specific T-cell responses in 84% patients	HR for PFS 0.23 in immune responders vs. non-responders

Abbreviations: OS, overall survival; mOS, median OS; AE, adverse event; PFS, progression-free survival; mPFS, median PFS; SD, stable disease; PD, progressive disease; ORR, objective response rate; HR, hazard ratio. Clinical outcomes are reported as defined in the original studies and vary across trials due to differences in study design and follow-up; endpoints were not standardized across studies.

### 3.3. Comparative Immunogenicity of Neoantigen Vaccine Types

As described above, neoantigen vaccines across multiple platforms, including peptide, mRNA and DNA, have demonstrated the capacity to induce robust immune responses. However, the magnitude and quality of these immune responses vary considerably across vaccine technologies, consistent with the characteristics specific to each vaccine type summarized in Table 3.

Peptide vaccines remain the most rapid and cost-efficient to manufacture owing to the simplicity of standardized chemical synthesis. In contrast, peptide-pulsed DC vaccines represent the most resource-intensive approach, requiring leukapheresis, ex vivo culture, antigen loading, and extensive quality control. Despite their strong induction of immune responses, the logistical complexity and limited scalability of peptide-pulsed DC vaccines may constrain their broad implementation in large patient populations, highlighting the practical trade-off between immunogenic potency and feasibility of large-scale clinical implementation. mRNA vaccines also require relatively long production timelines and formulation into lipid nanoparticles (LNPs) to achieve stability and efficient delivery. DNA vaccines typically involve longer production timelines because plasmid design, amplification, and purification are inherently time-consuming.

**Figure 1:**
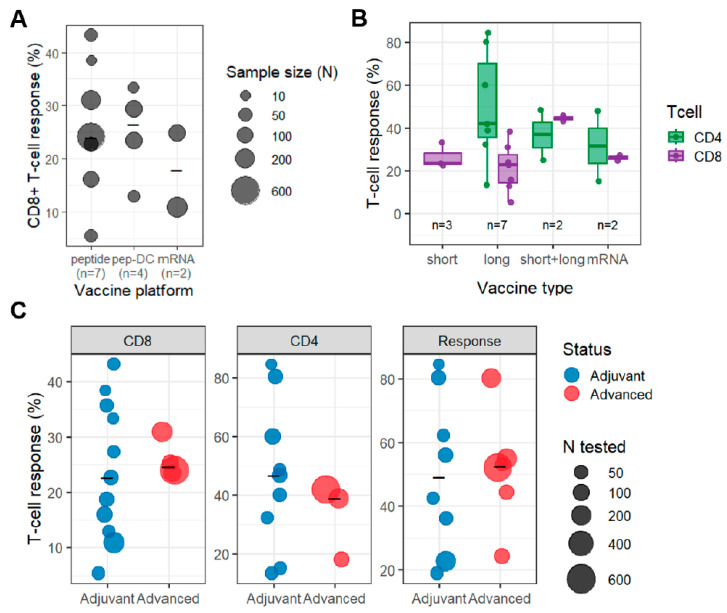
T-cell responses reported across neoantigen vaccine studies. All panels summarize T-cell responses based on administered neoantigen peptides. Each point represents one study, and bubble size indicates the number of peptides administered. The bar represents the average for each study. (**A**) Proportion of administered peptides inducing CD8^+^ T-cell responses across peptide, mRNA, and dendritic cell (DC) vaccine platforms. (**B**) CD4^+^ and CD8^+^ T-cell responses across short, long, and short + long peptide formulations and mRNA vaccines. (**C**) CD4^+^, CD8^+^ and overall T-cell responses in adjuvant and advanced disease settings.

Comparative analyses from multiple clinical studies, summarized in Figure 1A, show that peptide-based vaccines, including peptide-pulsed autologous DCs, tended to yield higher CD8^+^ T-cell response rates than mRNA-based vaccines, potentially reflecting differences in antigen processing. In mRNA-based vaccines, multiple neoantigen epitopes are typically encoded within a single construct, resulting in simultaneous intracellular expression and a tendency toward immunodominance during antigen processing, which may reduce detectable per-epitope CD8^+^ responses for individual targets despite broad epitope coverage. Across different vaccine types, more than 20% of the administered neoantigen peptides triggered detectable immune responses in most studies, with several cohorts exceeding 30%, indicating that all platforms are capable of inducing measurable antigen-specific immunity. Notably, peptide-pulsed autologous DCs consistently showed higher CD8^+^ T-cell responses, which may reflect more efficient class I antigen presentation and controlled epitope delivery. By contrast, in mRNA-based vaccines, antigen processing may preferentially amplify a limited subset of dominant epitopes rather than uniformly enhancing responses across all encoded targets. In addition, antigen delivery efficiency differs across platforms: peptide-based vaccines provide defined antigens directly to antigen-presenting cells, whereas mRNA vaccines depend on cellular uptake, translation, and downstream processing, which may further shape the magnitude and hierarchy of vaccine-induced T-cell responses. However, a larger fraction of neoantigens preferentially induced CD4^+^ rather than CD8^+^ T-cell responses, likely reflecting the use of long peptides and mRNA constructs that favor presentation via HLA class II pathways, even when targets were selected solely based on predicted HLA class I binding. This CD4^+^ bias may reflect both competition among multiple encoded epitopes and intrinsic antigen-processing pathways of endogenously translated proteins that favor HLA class II presentation. However, because the number of published mRNA vaccine cohorts remains limited compared with peptide-based platforms, direct numerical comparisons should be interpreted with caution, and no definitive conclusions regarding platform superiority can be drawn at this stage. Further studies with larger and more balanced cohorts will be required to enable robust comparative evaluation across vaccine modalities.

To contextualize these findings, we compiled CD8^+^ and CD4^+^ T-cell response data across studies, focusing on peptide-based formats (Figure 1B). CD4^+^ T cells can enhance CD8^+^ priming through licensing of antigen-presenting cells, provide cytokine support that sustains effector function, and promote epitope spreading over time. In several vaccine studies, vaccine-induced CD4^+^ responses were associated with broadening of the T-cell repertoire and the persistence of antigen-specific memory, suggesting that CD4^+^ immunity may serve as a key component of durable vaccine activity even when circulating CD8^+^ frequencies appear modest. The combined short- and long-peptide approach strongly elicited both CD8^+^ and CD4^+^ T-cell responses, while long-peptide-only approach induced weaker CD8^+^ T-cell responses. mRNA vaccines showed CD4^+^-dominant patterns similar to long peptides. Despite their somewhat lower CD8^+^ response rates, mRNA vaccines possess platform advantages, including efficient multi-epitope encoding and strong clinical momentum, which supports their expanding role in immunotherapy. Clinical observations outside oncology further suggest platform-level immunologic effects associated with mRNA vaccination. For example, SARS-CoV-2 mRNA vaccination within 100 days of ICI initiation, but not influenza vaccination, was associated with a doubling of 30-month survival compared with non-SARS-CoV-2 mRNA vaccine group (60% vs. 30% in NSCLC; 75% vs. <50% in melanoma; *p* = 0.0002 and 0.0048) [37]. While this observation does not demonstrate tumor-specific or antigen-specific efficacy, it suggests that mRNA vaccination may induce a non-antigen-specific immune activation. This broad immune activation underscores the need for careful and thorough evaluation of potential immune-related adverse events, when mRNA vaccines are combined with immunotherapies.

Large-scale immunopeptidome studies indicate that 20–40% of predicted HLA class I peptides undergo post-translational modification (PTM), including phosphorylation, methylation, glycosylation and ubiquitination [38]. Because peptide vaccines mostly rely on synthetic peptides, these modified epitopes can be intentionally incorporated into vaccine design, an advantage highlighted in Table 3. In contrast, mRNA or DNA platforms cannot readily reproduce such PTMs, representing a limitation when targeting neoantigens whose immunogenicity depends on specific modifications. The incorporation of PTMs may expand the range of targetable neoantigens for peptide-based platforms, although accurate PTM identification remains a major technical challenge.

### 3.4. Immune Conditions Across Treatment Setting

The effectiveness of neoantigen-based therapies is strongly influenced by the immune context in which they are administered. In particular, prior chemotherapy is expected to markedly impair host immune function, and therefore considering the treatment timing is important to evaluate vaccine-induced T-cell responses. In the report by Oyama et al., where adjuvant and advanced cohorts were evaluated under the same assay conditions, the adjuvant cohort showed higher neoantigen-specific T-cell responses in both peptide-level and patient-level analyses [18]. Peptide-level CD8^+^ and CD4^+^ response rates were 35.7% and 40.0% in the adjuvant group compared with 25.4% and 18.2% in advanced diseases. Patient-level response rates were likewise higher in the adjuvant cohort for both CD8^+^ and CD4^+^ T cells (100% vs. 67%). These observations likely reflect the cumulative detrimental effects of prior systemic therapies and chronic tumor burden on T-cell function, which may affect immune responsiveness in advanced settings. In contrast, patients treated in earlier disease stages generally maintain a more intact systemic immune milieu, providing a more favorable environment for vaccine-induced priming and expansion. Similarly, in our several clinical trials for peptide vaccine using multiple oncoantigens in esophageal cancer patients, CD8^+^ T-cell response rates were markedly higher in the adjuvant setting (98%) than in advanced disease (83%) [39,40,41]. Taken together, these data suggest that neoantigen-based vaccines may achieve greater therapeutic potential when administered in earlier treatment settings with lower tumor burden and less treatment-induced immune dysfunction, although cross-study comparisons remain limited by heterogeneity in vaccine platforms, study designs, and disease contexts (Figure 1C).

From an immunological standpoint, these observations align with a broader principle that effective cancer vaccination requires not only antigen specificity but also a permissive immune landscape. Earlier treatment settings are characterized by preserved antigen-presenting cell function, a more diverse naïve and memory T-cell repertoire, and reduced cumulative exposure to cytotoxic therapies. In contrast, advanced disease is frequently associated with chronic antigen stimulation, T-cell exhaustion, myeloid-driven immunosuppression, and impaired lymphoid architecture, all of which may constrain the capacity of vaccination to induce durable immunity. These considerations highlight why treatment timing should be regarded as an integral component of vaccine efficacy rather than a confounding variable alone.

## 4. Conclusions

Neoantigen vaccines are emerging as a versatile and scalable approach to personalized cancer immunotherapy, but several challenges must be addressed to fully unlock their therapeutic potential. In particular, tumor-intrinsic factors and the immunosuppressive tumor microenvironment remain major barriers that can limit the vaccine-induced antitumor immune responses. To address these challenges, flexible design of mRNA incorporating some immune-modulatory elements or by facilitating rational combination strategies with ICIs or other microenvironment-targeting therapies may induce effective neoantigen-related antitumor immune responses.

Future progress will require large-scale randomized clinical trials particularly in adjuvant and perioperative settings to validate survival benefits suggested by early-phase studies and to clarify optimal sequencing with standard treatments and immunotherapies. Advances in antigen prediction, including accurate modeling of HLA presentation, clonality, and post-translationally modified epitopes, will be essential to improving vaccine design. Equally critical will be the development of more efficient and rapid manufacturing pipelines across peptide, mRNA, and DNA vaccines to ensure timely vaccine delivery.

Overall, neoantigen vaccines offer a highly adaptable platform capable of targeting each patient’s unique tumor mutations. Importantly, continued integration of immunological, genomic, and clinical data will be required to translate immunogenicity into reproducible clinical benefit across diverse tumor types and patient populations. With continued technological and clinical advances, the prospect of reliably converting tumor-specific mutations into potent, safe, and durable antitumor immune responses is becoming increasingly realistic.

## Figures and Tables

**Table 1:** Published clinical studies of personalized neoantigen vaccines.

Author	Cancer Type	Vaccine Type	Number of Patients	Treatment Setting	Combination Therapy	Immune Response				Clinical Response				
						Tested	CD8^+^/ CD4^+^	CD8^+^	CD4^+^	CR	PR	SD	PD	Other Outcomes
Carreno, 2015 [5]	Melanoma	Short peptides (DCs)	3	Adjuvant	-	21	ND	33.3%	ND	1	0	2	0	
Ott, 2017 [6]	Melanoma	Long peptide	6	Adjuvant	-	97	ND	15.5%	59.8%	-	-	-	2	4/6 relapse-free at 25 mo; 2 had CR after anti-PD-1
Keskin, 2019 [7]	Glioblastoma	Long peptide	8	Adjuvant		37	19.0%	5.4%	13.5%	0	0	0	8	mPFS 7.6 mo; mOS 16.8 mo
Hilf, 2019 [8]	Glioblastoma	Long peptide	8	Adjuvant		13	84.6%	38.5%	84.6%	1	3	1	3	mPFS 14.5 mo; mOS 24.8 mo
Ott, 2020 [9]	Melanoma	Long peptide	27	Advanced	Nivolumab	570	52%	24%	42%	1	15	7	4	mPFS 23.5 mo; 12-mo OS 96%
	NSCLC	Long peptide	18	Advanced	Nivolumab		47%			0	7	9	2	mPFS 8.5 mo; 12-mo OS 83%
	Bladder cancer	Long peptide	15	Advanced	Nivolumab		52%			1	3	9	2	mPFS 5.8 mo; 12-mo OS 67%
Fang, 2020 [10]	Pan-cancer	Long peptide	22	Advanced	-	176	80.1%	ND	ND	0	0	15	7	mPFS 4.6 mo; mOS not reached
Chen, 2021 [11]	Pancreatic cancer	Long peptide	7	Advanced	-	70	44.3%	ND	ND	0	2	4	1	Mean PFS 3.1 mo; mean OS 24.1 mo
Morisaki, 2021 [12]	Pan-cancer	Short peptides (DCs)	17	Advanced	-	119	ND	23.5%	ND	1	3	11	2	
Awad, 2022 [13]	NSCLC	Long peptide	21	Advanced	Pembrolizumab	204	55%	31%	39%	0	11	9	1	mPFS 7.2 mo; mOS 20 mo
Zelba 2022, [14]	Breast cancer	Short + long peptide	4	Adjuvant	-	37	62.2%	43.2%	48.6%	-	-	-	-	No recurrence; RFS: 24–60 mo
Latzer, 2024 [15]	Glioblastoma	Short + long peptide	173	Adjuvant/ Advanced		-	-	-	-	-	-	-	-	mOS 31.9 mo
Morisaki, 2024 [16]	Breast cancer	Short + long peptides (DCs)	5	Adjuvant	-	31	ND	12.9%	32.3%	-	-	-	-	
Braun, 2025 [17]	Renal cell carcinoma	Long peptide	9	Adjuvant	Ipilimumab	129	47.3%	-	-	-	-	-	-	No recurrence at 40.2 mo
Oyama, 2025 [18]	Pancreatic cancer	Short + long peptides (DCs)	7	Adjuvant	-	47	36.2%	35.7%	40.0%	-	-	-	-	6/7 recurrence-free at 61 mo
			9	Advanced	-	78	24.4%	25.4%	18.8%	-	-	-	-	Better OS in responders vs. in non-responder
Sahin, 2017 [19]	Melanoma	mRNA	13	Adjuvant/ Advanced	-	125	76.0%	24.8%	48.0%	0	0	0	5	Tumor regressions in advanced patients; no serious AEs
Rojas, 2023 [20]	Pancreatic cancer	mRNA	16	Adjuvant	Atezolizumab + Chemotherapy	230	ND	10.9%	ND	-	-	-	-	RFS 18 mo; grade ≥3 AEs in 6% patient
Weber, 2024 [21]	Melanoma	mRNA	157	Adjuvant	Pembrolizumab	-	-	-	-	-	-	-	-	18-mo RFS 79% (vs. 62% control); grade ≥3 AEs in 12% patients
Ingels, 2024 [22]	NSCLC	mRNA (DCs)		Adjuvant	-	33	42.4%	27.3%	15.2%	-	-	-	-	50% (3/6) recurrence within 2 yr
Zhang, 2024 [23]	Breast cancer	DNA	18	Adjuvant	-	45	31.1%	22.2%	11.1%	-	-	-	-	36-mo RFS 87.5%; no grade 3 AEs
Yarchoan, 2024 [24]	Hepatocellular carcinoma	DNA	36	Advanced	Pembrolizumab	248	64.0%	-	-	3	8	9	14	No grade 3 AEs

Abbreviations: CR: complete response, PR: partial response, SD: stable disease, PD: progression disease, mPFS: median progression-free survival, OS: overall survival, mOS: median OS, RFS: relapse-free survival, AE: adverse event. Clinical outcomes are reported as defined in the original studies and vary across trials due to differences in study design and follow-up; endpoints were not standardized across studies.

**Table 3:** Comparison of advantages and limitations in each vaccine type.

Feature	Peptide	Peptide-Pulsed DCs	RNA	DNA
Immunogenicity	++	+++	++	+
Safety (low toxicity)	+++	+++	+	++
Cost	+++	−	−	++
Rapid manufacturing	+++	− (requires cell manufacturing)	+	+
Multi-epitope encoding	+ (possible, but increase cost)	+ (possible, but increase cost)	+++ (but may occur immunodominance)	+++
Post-translational modification compatibility	+++	+++	−	−
Stability/storage	++	± (frozen/short-term)	± (frozen storage needed)	++
Delivery complexity	++	− (ex vivo cell manufacturing)	± (LNP needed)	−
Scalability	++	− (limited by cell manufacturing)	++	++
Clinical momentum	+	±	+++ (accelerated by SARS-CoV-2 vaccine)	−

+, ++, and +++ indicate increasing degrees of advantage, whereas − indicates a disadvantage.

## Data Availability

No new data were created or analyzed in this study. Data sharing is not applicable to this article.
